# Ameliorative effect of carveol on scopolamine-induced memory impairment in rats 

**DOI:** 10.22038/IJBMS.2022.66797.14647

**Published:** 2022-12

**Authors:** Komal Latif, Saneela Saneela, Arif-ullah Khan

**Affiliations:** 1 Riphah Institute of Pharmaceutical Sciences, Riphah International University, Islamabad

**Keywords:** Alzheimer, Amnesia, Anti-inflammatory, Anti-oxidant, Carveol, Memory impairment, Neuroinflammation

## Abstract

**Objective(s):**

*Carveol* is a naturally occurring terpenoid with antispasmodic, carminative, astringent, indigestion, and dyspepsia properties, as well as anti-diabetic, anti-oxidant, anti-hyperlipidemia, and anti-inflammatory properties in the liver. Research also suggests that it has memory-enhancing and anti-oxidant properties. The purpose of this research was to see whether carveol could protect rats against scopolamine-induced memory loss in a rat model.

**Materials and Methods:**

Thirty male Sprague-Dawley rats (200–250 g) were grouped as the saline group receiving saline, disease group receiving scopolamine, and four treatment groups among which three groups received scopalamine+carveol and one group received scopalamine+donepezil for 28 days. Followed by *in vitro*, behavioral, anti-oxidant, and molecular studies were done. *P*<0.005 was considered statistically significant.

**Results:**

The in vitro assay showed that carveol caused diphenyl-1-picrylhydrazyl inhibition. In-vivo findings revealed that carveol (50, 100, and 200 mg/kg) significantly improved dementia by reducing escape latency and spending more time in the targeted quadrant in the Morris water maze test. Increased number of entries and percent spontaneous alterations were observed in rats’ Y-maze test. In animal brain tissues, i.e., cortex and hippocampus, carveol enhanced glutathione, glutathione-s-transferase, catalase, and reduced lipid peroxide levels. Carveol also improved cellular architecture in histopathological examinations and decreased expression of inflammatory markers such as amyloid-beta, nuclear factor kappa light chain activated B cells, tumor necrosis factor-alpha, cyclooxygenase 2, prostaglandin E2, and interleukin-18, as evidenced by immunohistochemistry and enzyme-linked immunosorbent assays, as well as molecular investigations.

**Conclusion:**

This study suggests that the compound could be potent against amnesia mediated through anti-oxidant, amyloid-beta inhibition, and anti-inflammatory pathways.

## Introduction

Alzheimer’s disease (AD), schizophrenia, epilepsy, and depression all have cognitive loss as common characteristics (1, 2). AD is a multifactorial neurodegenerative disease that often begins slowly and is progressively fatal (3, 4). Risk factors for the development of AD include hypertension, hyperlipidemia, heart disease, and diabetes (5). In 2015, a report estimated about 46.8 million people with dementia cases worldwide (6, 7). As the prevalence of its number is continually growing, it is expected to double every 20 years, 74.7 million people are expected to be affected by AD 2030 (8). Low-middle-income nations now account for 58 percent of the world’s aging population with dementia, with the prevalence projected to rise to 68 percent by 2050 (9). There is a high prevalence, due to the percentage of elderly people increasing more quickly, in populous nations like China, Pakistan, and their South Asian and Western Pacific neighbors (10). Pakistan is the world’s sixth most populous nation, with an estimated percentage of dementia cases of 150,000–200,000 people (11).

AD is characterized by a psychological and behavioral disturbance that ends in the loss of memory, impairment of cognition, plaques of amyloid-beta (Aβ) protein, and amyloid accumulation of 39-42 AA peptides (12). Deposition of the amyloid precursor protein, neuroﬁbrillary tangles, and abnormal tau protein filaments deposition in the form of intraneuronal neurofibrillary tangles generate free radicals which ultimately cause synaptic dysfunction, neurotoxicity, and neuroinflammation (13). An abnormal decrease in the levels of acetylcholine in the brain and phosphorylation of tau proteins deteriorates forebrain and cholinergic neurons disrupt the neuronal pathways and neurotransmitters involved in the process of learning and memory (14). In neurodegenerative illnesses such as Alzheimer’s disease, Parkinson’s disease, and ischemic brain damage, synaptic disruption and excessive generation of oxidative stress indicators reduce memory capacity (15, 16). In these neurodegenerative diseases, disturbances in metabolic pathways such as loss of ionic gradient, release of excitatory neurotransmitters, and production of harmful radicals all lead to death (17). Scopolamine, a muscarinic cholinergic receptor antagonist, is an effective pharmacological drug for creating a partial amnesia model (18). As a result, scopolamine may be useful in both animal models of amnesia and in defining prospective cognition-enhancing medicines (19). 

Carveol is a naturally occurring terpenoid found in Mentha spicate and Carum carvi essential oils (20, 21). Carveol has been used for antispasmodic, carminative, astringent, indigestion, and dyspepsia in traditional Chinese medicine (22). Its anti-diabetic, anti-oxidant, anti-hyperlipidemia, and anti-inflammatory properties in the liver are also shown (23). The current study aims to investigate the anti-amnesic potential of carveol, employed* in vitro, in vivo*, and in molecular techniques.

## Materials and Methods

***Chemicals***

Carveol, donepezil, and scopolamine hydrobromide were purchased from Sigma (St. Louis, USA). Dimethyl sulfoxide (DMSO) was purchased from Merck Millipore (Billerica, MA, USA). Antibodies (anti-A, anti-NF-B, anti-TNF-, and anti-COX-2) from rats, 3, 3-diaminobenzidine peroxidase (DAB), Avidin-Biotin Complex (ABC), 1-chloro-2,4-dinitrobenzene (CDNB), hydrogen peroxide (H_2_O_2_), trichloroacetic acid (TCA), formalin, thiobarbituric acid (TBA) were purchased from St. Louis, MO, USA. The secondary antibody was obtained from Abcam in the United Kingdom. Rat Aβ, rat NF-κB, rat TNF-α, rat IL-18, and rat PGE2 ELISA kits were obtained from Elabscience China.

***Animals***

In this experiment, male Sprague-Dawley rats weighing 200–250 g were utilized and kept in the animal house of Riphah Institute of Pharmaceutical Sciences (RIPS) in Islamabad (n=30, 5 in each group). Experiments were carried out with the approval of RIPS’ Research and Ethics Committee (Ref. No. REC/RIPS/2020/14) and in accordance with the Principles of Laboratory Animal Care. A 12/12 hr light-dark cycle and relative humidity at 55% ± 5% were maintained, and the room temperature was controlled at 25 °C ± 1 °C. *Ad libitum* food and water were available.

***Study design***

Rats were divided into 6 groups. 

Group 1: Saline group received 10 ml/kg normal saline containing 5% DMSO intraperitoneal (IP). 

Group 2: Disease group received five injections of Scopolamine (1 mg/kg, IP) dissolved in a mixture of normal saline and 5% DMSO for 28 days.

Group 3: Treatment group received Scopolamine (1 mg/kg) daily, followed by carveol (50 mg/kg) IP for 28 days.

Group 4: Treatment group received scopolamine (1 mg/kg) daily, followed by carveol (100 mg/kg) IP for 28 days.

Group 5: Treatment group received scopolamine (1 mg/kg) daily, followed by carveol (200 mg/kg) IP for 28 days.

Group 6: Treatment group received scopolamine (1 mg/kg) daily, followed by donepezil (5 mg/kg) IP for 28 days.

**Figure 1:**
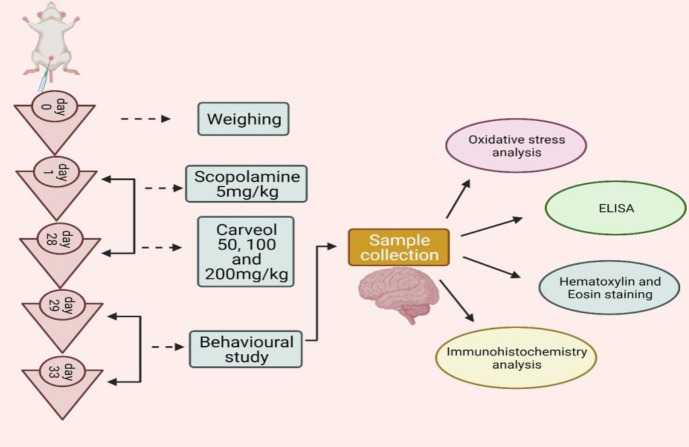
Schematic diagram of the experiment protocol

Two hr after the scopolamine injection, carveol and donepezil were dissolved in mixture of normal saline and 5% DMSO and injected intraperitoneally. Rats were taught for the Morris water maze test on the 29th, 30th, and 31^st ^days, and a probe test was performed on the 32nd day to evaluate spatial learning. On day 33, we conducted three trials of the Y-maze test for memory impairment and observed spontaneous behavior change. On the same day, all groups of rats were terminally anesthetized with sodium pentobarbital (60 mg/kg, IP) before being decapitated. Tissues were collected and centrifuged in phosphate-buffered saline (0.1 M PBS), pH 7.4, 5% w/v. Using a micropipette, the supernatant was collected and stored at -80 °C. (n=5/group) Brain tissues were extracted and subjected to anti-oxidant and molecular testing, and maintained in 4 percent formaldehyde for paraffin block production to process and analyze Hematoxylin and Eosin (H&E) and immunohistochemistry (IHC) examination. The experimental protocol is described in Figure 1.

***DPPH free radical scavenging assay***

The ability of the compounds to scavenge free radicals was tested using 1,1-diphenyl, 2-picrylhydrazyl (DPPH). Different dilutions (1.25, 2.50, 5, 10, 20 μg/ml) of compounds (0.1 ml) were added to 0.004 % methanolic solution of DPPH. A UV spectrophotometer was used to measure the absorbance at 517 nm after 30 min. Ascorbic acid was utilized as positive control.

The scavenging activity was measured as a percentage:

$\left(\frac{A_{0}-A_{1}}{A_{0}}\right)\times 100$

A1 is the absorbance of the chemical sample, while Ao is the absorbance of the control. Inhibition curves were generated using the GraphPad Prism software (GraphPad, San Diego, California, USA) to obtain the values of median inhibitory concentrations in each experiment. (IC_50_) (24). 

***Morris water maze (MWM) test***

The standard technique outlined by elements of learning and memory in rats is utilized in conjunction with the MWM test [14]. This experiment was carried out in a big circular tank (120 cm diameter x 50 cm height, with a colored round platform 1 cm below the water’s surface) with the platform fixed for a visual cue. To estimate the escape latency time (time to locate hidden platform), rats were taught to identify the hidden platform with a maximum latency time of 180 seconds at the start of the experiment, and swim distance was recorded in each trail across three trail days for three consecutive days. During the probe test, each rat was dropped without a platform opposite to the targeted quadrant, and time spent in the targeted quadrant was measured by using a stopwatch (22, 25).

***Y-maze test***

The y-maze was conducted in a y-maze device that had three identical arms, each measuring 40 cm long, 35 cm high, and 12 cm broad. Rats were put in the arm’s middle and permitted to freely roam throughout the labyrinth. To calculate spontaneous changes, five sessions were conducted. The Y-maze spontaneous alteration was characterized as overlapping triplet sets of consecutive entries into each of the three arms (26).

***Determination of anti-oxidant profile***

*GSH and GST assay*

Freshly extracted brain tissue samples were homogenized (0.1 M PBS, pH 7.4), mixed with phenyl methyl sulfonyl fluoride (PMSF), and centrifuged (4000 x g) for 10 min at 4 °C. The GSH levels were determined by collecting the supernatant with a micropipette and using the previously described technique with minor changes [16]. To dissolve 0.6 mM DTNB, 2 ml of the following mixture was combined with 0.2 ml of previously collected supernatant in 0.2M PBS. To make up the final volume and collect 3 ml of solution, 0.1 M PBS was utilized. After 10 min, the absorbance was measured at a wavelength of 412 nm. PBS and DTNB solutions were used to adjust the absorbance values for the negative and positive controls, respectively. The GSH levels were calculated and represented in mol/mg of proteins. The GST level was similarly determined using a well-established technique with just minor changes [17]. A test solution containing 5 mM GSH and 1 mM CDNB was made with freshly produced PBS (0.1 M). In duplicate, 60 L of supernatant was added to a glass vial containing 1.2 ml of test solution. To make the blank solution in triplicate, distilled water was added in the same quantity. The absorbance of these prepared solutions (210 l) was measured using an ELISA microplate reader (max = 340 nm, 5 min at 23 °C). Total protein concentration was measured using a BCA kit according to the manufacturer’s instructions, and the resulting GST values were represented in mol/mg of protein.

*Catalase assay*

The activity of catalase was determined by mixing 3 ml H_2_O_2_ with 0.05 ml tissue supernatant. At 240 nm, the absorbance activity was measured against a blank containing just 3 ml of PBS. The absorbance is proportional to the amount of H_2_O_2_ present, which is reduced when catalase destroys H_2_O_2_. This is a measurement of H_2_O_2_ breakdown and is given as mol H_2_O_2_ decomposed per milligram of protein per minute (27).

*LPO Assay*

Another important oxidative stress marker is LPO, which uses a colorimetric method to detect TBARS (thiobarbituric acid reactive compounds) (28). Each rat’s cortical and hippocampal tissues were homogenized separately in 10 ml of 20 mM Tris-HCl at 4 °C in a Polytron homogenizer while maintaining a pH of 7.4. The supernatant was recovered after centrifuging the homogenate at 1000 g for 10 min at 4 °C. A new ferric ammonium sulfate solution was made. The collected supernatant received 40 microliters of the aforementioned solution, which was then incubated at 37 °C for 30 min. Then, 400 mg of 2-thiobarbituric acid (TBA) was dissolved in 50 ml of water to make a 0.8 percent solution. TBA was added to the supernatant and ferric ammonium sulfate mixture in a total of 75 liters. A plate reader was used to detect absorbance at a wavelength of 532 nm.

***Hematoxylin and Eosin (H&E) staining***

After deparaffinizing tissue slides with xylene (100 percent), slides are rehydrated. Then slides were rinsed in PBS and immersed in hematoxylin for a minimum of 10 min. The slides were then dehydrated by treating them with successive ethanol dilutions of 70, 95, and 100 percent, followed by xylene fixation and application of coverslips (28). Microscopic pictures were examined using an Olympus light microscope (Olympus, Japan) and ImageJ NIH software. Each group received 5 microscopic pictures to evaluate neuron shape, cellular infiltration, and vacuole development.

***Immunohistochemistry (IHC) investigation***

The slides were deparaffinized and rehydrated before being treated with proteinase K to extract antigen. The slides were then washed in 0.1 M PBS before being immersed in 3 percent H_2_O_2_ for ten minutes to suppress peroxidase activity. Again slides were washed in 0.1 M PBS and incubated in a humidified room for at least one hour with normal goat serum (5 percent NGS with 0.1 percent Triton X-100). After treating slides with primary antibodies such as anti-Aβ, anti-NF-κB, anti-TNF-α, and anti-COX-2, they were incubated overnight at 4 °C (Dilution 1:100, Santa Cruz Biotechnology, USA). The following day, the slides were washed twice with 0.1 M PBS before being incubated in a humidified room for one and a half hours with biotinylated secondary antibodies (dilution factor 1:50). After another wash with 0.1 M PBS, the slides were incubated in a humidified room for one hour with ABC. Finally, slides were stained with DAB, washed with water, and dehydrated, followed by xylene fixation and placement of coverslips following application of the mounting medium. A light microscope was used to acquire three microscopic pictures for each slide (Olympus, Japan). ImageJ was used to evaluate Aβ and NF-κB expression, and relative integrated density was computed (29).

***Enzyme-linked immunosorbent assay***

The Aβ, NF-κB, TNF-α, IL-18, and PGE2 ELISAs were performed according to the manufacturer’s instructions. Following homogenizing a sufficient amount of brain tissues (50 mg), the supernatant was recovered after centrifugation (at 4000 x g for 30 min). The total protein concentration in each group was determined using the BCA method. Using a 96-well plate, protein samples were treated with antibodies supplied in the kit, and absorbance values were measured using a microplate reader. Concentrations in picograms per liter (pg/ml) were then adjusted to total protein content in pg/mg total protein. All steps were repeated three times (30).

***Statistical analysis***

Image J was used to evaluate the morphological data (9). The data is given as a mean with a standard error of the mean (31). Graph pad prism 6 was used to apply two-way ANOVA with *post hoc* Tukey’s test. *P*<0.005 was deemed statistically significant. 

## Results

***Effect of carveol on DPPH free radicals scavenging inhibition***

**Figure 2:**
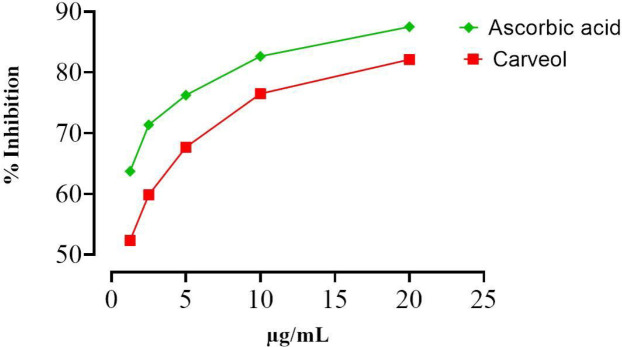
Anti-oxidant potential of carveol using the DPPH assay

In DPPH free radical scavenging assay, increase in carveol concentrations, i.e., 1.25, 2.5, 5, 10, and 20 µg/ml showed increased DPPH inhibition of 52.35 ± 0.45, 59.85 ± 0.56, 67.68 ± 0.45, 76.50 ± 0.14, and 82.12± 0.26, respectively. At the same concentrations, ascorbic acid showed DPPH inhibition of 63.72±0.51, 71.37±0.69, 76.25±0.55, 82.65±0.70, and 87.51±0.62% as shown in Figure 2.

***Effect of carveol on cognitive impairment***

**Figures 3a, b, and c:**
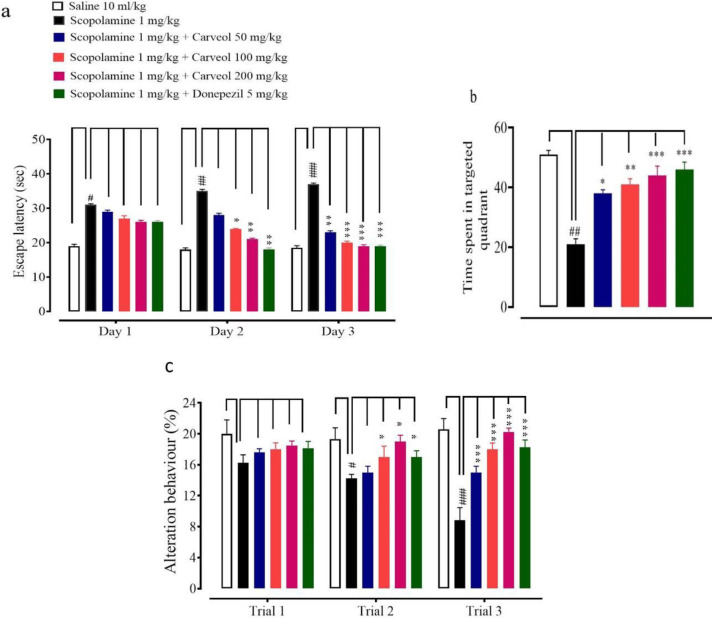
Effects of carveol and donepezil on escape latency time of rats on days 1, 2, and 3 in Morris water maze test, time spent in the targeted quadrant and alteration behavior of rats on trials 1, 2, and 3 in the Y-maze test. Values expressed as mean ± SEM (n=5). Two-way ANOVA with *post hoc* Tukey's test. #*P*<0.005, ##*P*<0.001, ###*P*<0.001 vs saline group, *P<0.05, **P<0.01, ***P<0.001 vs scopolamine group

In the MWM test, the escape latency time for the carveol was measured in three trials per day for three consecutive days. In the saline (10 mg/kg) group, escape latency at days 1, 2 and 3 was 19.0 ± 0.5, 18 ± 0.5, and 18.5 ± 0.6 sec, respectively. In the scopolamine (5 mg/kg) group, escape latency increased to 31.0 ± 0.3, 35.0 ± 0.5, 37.0 ± 0.3 sec, respectively. In the carveol (50 mg/kg) treated group, escape latency decreased to 29.0 ± 0.5, 28.0 ± 0.5, 23.0 ± 0.4 sec, respectively. In carveol (100 mg/kg) escape latency reduced to 27.0 ± 0.81, 24.0 ± 0.14, 20.0 ± 0.43 sec, respectively. In carveol (200 mg/kg) escape latency further reduced to 26.0 ± 0.5, 21.0 ± 0.3, 19.0 ± 0.9 sec, respectively. In donepezil (5 mg/kg) escape latency was 26.0 ± 0.4, 19.0 ± 0.5, 18 ± 0.3 sec, respectively vs scopolamine group as shown in Figure 3a. On the 4^th^ day the duration spent in the desired quadrant by the scopolamine 5 mg/kg group was significantly lower, i.e., 21.0 ± 2 sec vs saline group 51.0 ± 1 sec, Figure 3b. The observed time spent in the target quadrant of carveol 50, 100, 200 mg/kg, and donepezil 5 mg/kg were significantly higher, i.e., 38 ± 1, 41 ± 2, 44 ± 1, and 46 ± 1 sec vs the scopolamine group.

***Effects on alteration behavior***

The Y-maze task was performed to analyze the spatial working memory using spontaneous alternation behavior (%). In the saline (10 mg/kg) group, spontaneous alterations at trials 1, 2, and 3 were 20 ± 1.80, 19.32 ± 1.45, 20.58 ± 1.40, respectively. In scopolamine (5 mg/kg) group, spontaneous alterations reduced to 16.25 ± 1.04, 14.27 ± 0.43, 8.85 ± 1.39, respectively. In carveol (50 mg/kg) treated group, spontaneous alterations increased to 17.62 ± 0.38, 15 ± 0.71, 15 ± 0.8, respectively. In carveol (100 mg/kg) treated group, spontaneous alterations enhanced to 18.03 ± 0.71, 17 ± 1.22, 18 ± 0.71, respectively. In carveol (200 mg/kg) treated group, spontaneous alterations further increased to 18.5 ± 0.5, 19 ± 0.70, 20 ± 0.43, respectively. In donepezil (5 mg/kg) treated group, spontaneous alterations were 18.15 ± 0.75, 17 ± 0.71, 18.25 ± 0.82 (Figure 3c). 

***Effect on oxidative stress markers***

**Figure 4:**
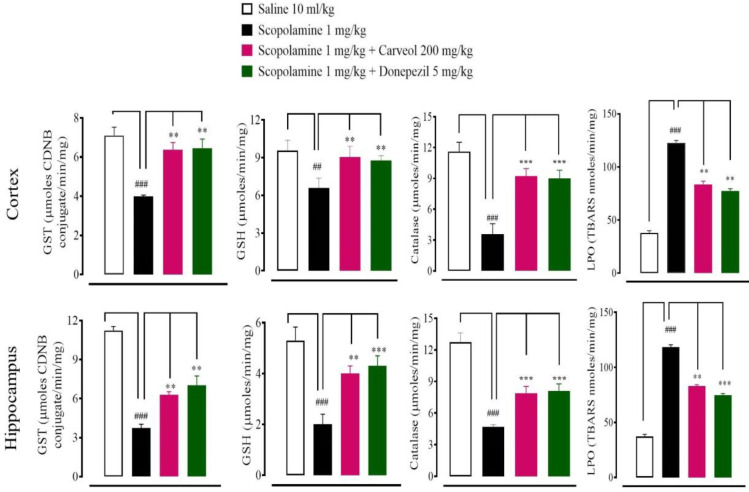
Effect of carveol and donepezil against reduced glutathione (GSH), glutathione-S-transferases (GST), catalase, and lipid peroxidation (LPO) in scopolamine-induced amnesic rat’s cortex and hippocampus tissues. Data presented as mean ± SEM (n=5). Two-way ANOVA with *post hoc* Tukey’s test. ##*P*<0.01, ###*P*<0.001 vs saline group, **P<0.01, ***P<0.001 vs scopolamine group

**Figure 5:**
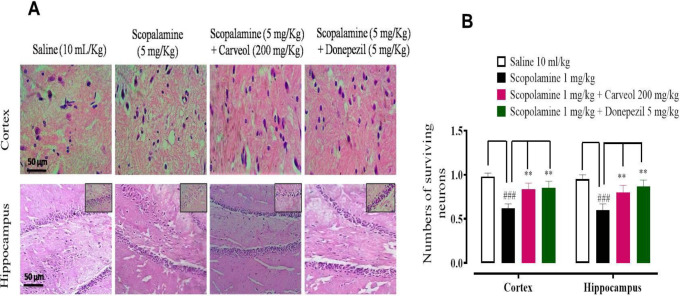
A and B represent the effect of carveol and donepezil against surviving neuron expression in rat’s cortex and hippocampus tissues, using the hematoxylin and eosin histopathological staining technique. Bar 50 µm, magnification 40x. Values expressed as mean ± SEM (n=5). Two-way ANOVA with *post hoc* Tukey’s test. ###*P*<0.001 vs saline group, **P<0.01, ***P<0.001 vs scopolamine group

In saline (10 ml/kg) group, GSH, GST, catalase, and LPO levels in cortex tissues were 9.56 ± 0.41, µmoles/mg, 7.10 ± 0.43 µmoles CDNB conjugate/min/mg, 11.61 ± 0.45 µmoles H2O2/min/mg, and 37.9 ± 2 nmoles/min/mg, respectively. In scopalamine (5 mg/kg) group GSH, GST, and catalase levels decreased to 6.57 ± 0.39 µmoles/mg, 3.99 ± 0.07 µmoles CDNB conjugate/min/mg, and 3.57 ± 0.51 µmoles H_2_O_2_/min/mg, and LPO level increased to 122.78 ± 2.10 nmoles/min/mg, respectively. In carveol (200 mg/kg) treated group GSH, GST, catalase levels increased to 8.77 ± 0.19 µmoles/mg, 6.37 ± 0.37 µmoles CDNB conjugate/min/mg, and 9.23 ± 0.36 µmoles H_2_O_2_/min/mg, and LPO level decreased to 83.57 ± 3 nmoles/min/mg, respectively. In donepezil (5 mg/kg) treated group GSH, GST, and catalase levels raised to 8.77 ± 0.19 µmoles/mg, 6.46 ± 0.46 µmoles CDNB conjugate/min/mg, and 9.0 ± 0.40 µmoles H_2_O_2_/min/mg, and LPO level reduced to 77.48 ± 2.10 nmoles/min/mg, respectively (Figure 5). In saline (10 ml/kg) group GSH, GST, catalasen, and LPO levels in hippocampus tissues were 5.3 ± 0.5 µmoles/mg, 11.23 ± 0.31 µmoles CDNB conjugate/min/mg, 12.72 ± 0.44 µmoles H_2_O_2_/min/mg, and 37.23 ± 2 nmoles/min/mg, respectively. In scopalamine (5 mg/ Kg) group GSH, GST, and catalase levels decreased to 2 ± 0.4 µmoles/mg, 3.73 ± 0.30 µmoles CDNB conjugate/min/mg, and 4.71 ± 0.10 µmoles H_2_O_2_/min/mg, and LPO level was increased to 118.4 ± 2.10 nmoles/min/mg, respectively. In carveol (200 mg/kg) treated group GSH, GST, and catalase levels increased to 4 ± 0.3 µmoles/mg, 6.28 ± 0.25 µmoles CDNB conjugate/min/mg, and 7.90 ± 0.32 µmoles H_2_O_2_/min/mg, and LPO level decreased to 83.15 ± 1.05 nmoles/min/mg, respectively. In donepezil (5 mg/kg) treated group GSH, GST, and catalase level raised to 4.3 ± 0.4 µmoles/mg, 7.01 ± 0.73 µmoles CDNB conjugate/min/mg, and 8.11 ± 0.33 µmoles H_2_O_2_/min/mg, and LPO level reduced to 74.62 ± 1.50 nmoles/min/mg, respectively (Figure 4).

***Histopathological examination***

The saline (10 ml/kg) group revealed no distinct pathological changes in cortex and hippocampus tissues. The scopolamine (1 mg/Kg) group showed vigorous histological changes in the cortex and hippocampus tissues of the rat’s brain. Abnormal histological changes were shown in the scopolamine group which included altered morphology of neurons comprising abnormal changes in cell size, shape (i.e., swollen, irregular angled, vacuolated), and abnormal staining (i.e., cytoplasmic eosinophilia and nucleus basophilia) in the cortex and hippocampus tissues. Carveol (200 mg/kg) and donepezil (5 mg/kg) attenuated infarct and damaged cells (Figure 5).

***Effect of carveol on neuroinflammation***

**Figure 6:**
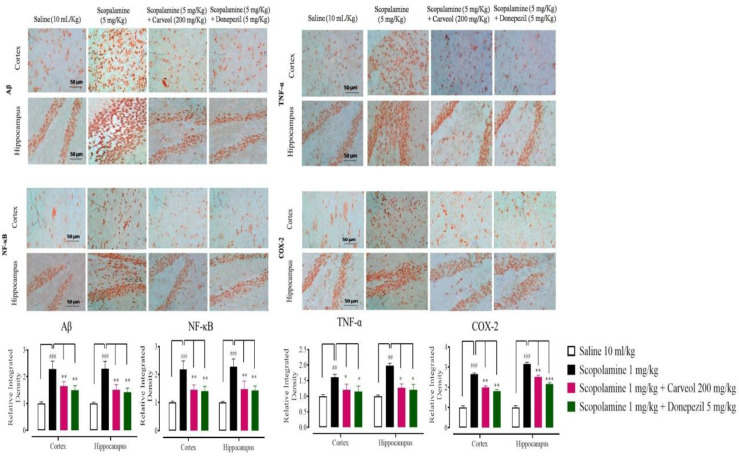
Inhibitory effects of carveol and donepezil against nuclear factor kappa Beta (NF-κB) expression in rats’ cortex and hippocampus tissues, using the immunohistochemical technique. Bar 50 µm, magnification 40x. Values expressed as mean ± SEM (n=5). Two-way ANOVA with *post hoc* Tukey’s test. ###*P*<0.001 vs saline group, **P<0.01, ***P<0.001 vs scopolamine group

**Figure 7:**
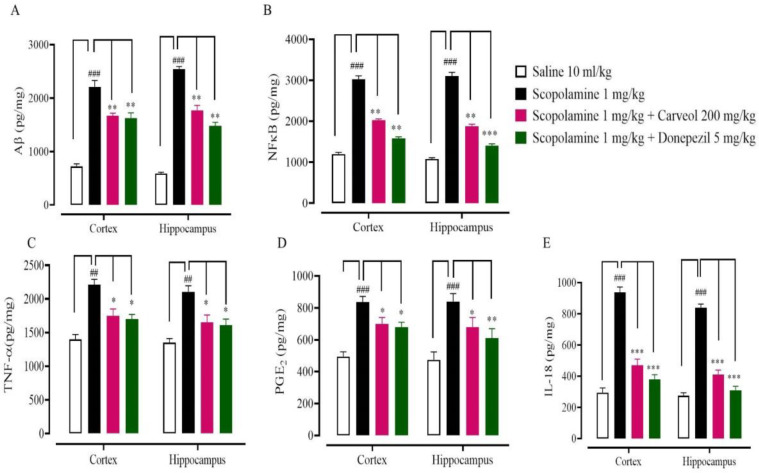
Inhibitory effect of carveol and donepezil against amyloid-beta (Aβ), nuclear factor kappa beta (NF-κB), tumor necrosis factor-alpha (TNF-α), interleukin-18 (IL-18), and prostaglandin E2 (PGE2) concentrations in rat’s cortex and hippocampus tissues, using enzyme-linked immunosorbent assay technique. Values expressed as mean ± SEM (n=5). Two-way ANOVA with *post hoc* Tukey’s test. ###*P*<0.001 vs saline group, **P<0.01, ***P<0.001 vs scopolamine group

To identify the significant involvement of inflammatory mediators in neuroinflammation induced by scopolamine, IHC was performed. Results revealed that the scopolamine (1 mg/kg) group revealed overexpression of Aβ, NF-κB, TNF-α, and COX-2 markers compared with the saline group in cortex and hippocampus tissues (Figure 5, ^##^*P*<0.001 and ^###^*P*<0.0001). Carveol (200 mg/kg) and donepezil (5 mg/kg) treated groups down-regulated the expressions of Aβ and NF-κB, TNF-α, and COX-2 in cortex and hippocampus tissues, relative to the scopolamine group (Figure 6). For further validation, ELISA was performed and the results revealed that in the saline (10 ml/kg) group Aβ, NF-*κ*B, TNF-α, IL-18, and PGE2 levels in cortex tissues were 720.33 ± 49.08,1200 ± 40, 1400 ± 70, 295 ± 30, and 495 ± 30 pg/mg, respectively. In the scopolamine (5 mg/kg) group Aβ, NF-*κ*B, TNF-α, IL-18, and PGE2 concentration increased to 2210 ± 119.3, 3030 ± 80, 2210 ± 80, 937 ± 35, and 837 ± 35 pg/mg, respectively. In the carveol (200 mg/kg) treated group Aβ, NF-*κ*B, TNF-α, IL-18, and PGE2 production decreased to 1667.24 ± 49.90, 2030 ± 30, 1750 ± 100, 470 ± 40, and 700 ± 40 pg/mg, respectively. In the donepezil (5 mg/kg) treated group Aβ, NF-*κ*B, TNF-α, IL-18, and PGE2 levels reduced to 1626.9 ± 95.37, 1580 ± 40, 1699 ± 70, 380 ± 30, and 680 ± 30 pg/mg, respectively. In the saline (10 ml/kg) group, Aβ, NF-*κ*B, TNF-α, IL-18, and PGE2 concentration in hippocampus tissues were 587.17 ± 25.03, 1080 ± 30, 1350 ± 60, 274 ± 20, and 840 ± 50 pg/mg, respectively. In scopolamine (5 mg/kg) group Aβ, NF-*κ*B, TNF-α, IL-18, and PGE2 production increased to 2537.1 ± 52.4, 3105 ± 90, 2105 ± 90, 840 ± 23, and 840 ± 50 pg/mg, respectively. In carveol (200 mg/kg) treated group Aβ, NF-*κ*B, TNF-α, IL-18, and PGE2 concentration decreased to 1763.84 ± 98.78, 1880 ± 50, 1650 ± 110, 410± 30, and 680 ± 60 pg/mg, respectively. In donepezil (5 mg/kg) treated group Aβ, NF-*κ*B, TNF-α, IL-18, and PGE2 production declined to 1480 ± 65.32, 1400 ± 50, 1610 ± 90, 310 ± 25, and 610 ± 60 pg/mg respectively (Figure 7).

## Discussion

The impact of carveol on scopolamine-induced memory impairment in rats was studied *in vitro, in vivo*, and through biochemical and molecular tests. Scopolamine, a nonselective centrally acting muscarinic receptor antagonist, has long been recognized to impair learning and memory in rats and humans (19, 32). This memory impairment experimental paradigm has been widely utilized in research to find medicines with potential therapeutic benefits in dementia (33, 34). 

Carveol’s anti-oxidant capacity was first assessed *in vitro* using the DPPH free radical scavenging test, which revealed that it had greater anti-oxidant potential and was then investigated *in vivo*.

On behavioral tests, scopolamine reduced cognitive function. In order to test this effect, rats were given intraperitoneal injections of scopolamine to cause memory impairments. The MWM and Y-maze tests were used in behavioral research to look into their learning and memory abilities, while biochemical experiments were used to look into their potential molecular processes (35). In the current study, scopolamine-injected rats showed an increase in escape and latency when compared with the control groups. Similarly, as compared with the scopolamine-injected rats, the animals given carveol plus donepezil had a shorter escape latency. Carveol showed neuroprotective benefits against scopolamine-induced memory deficits, according to the results of behavioral experiments. The number of entries and spontaneous alternation behavior were used to measure spatial working memory, which is reliant on the hippocampus (36, 37). Increased cognitive performance is associated with a greater proportion of spontaneous modification behavior (38).

Carveol exhibited dose-dependent enhanced spontaneous alteration behavior and numerous entrances of rats in the Y-maze test in contrast to scopolamine-treated rats in the current research, indicating higher cognitive activities.

Scopolamine causes the deposition of A42 and A40, which are the end products of -secretase amyloid precursor protein, which is produced by the cleaving of enzyme 1 (BACE1) (39). Increased BACE1 levels cause oxidative stress by activating JNK and p38 (40). Oxidative stress causes high oxygen consumption in the central nervous system, which contributes to AD pathogenesis (41, 42). According to previous research, oxidative stress is one of the first steps in the pathophysiology of memory loss (43). In the scopolamine group, oxidative damage was detected by low levels of GSH, GST, and catalase, and high levels of LPO, compared with carveol and donepezil, which had higher levels of GSH, GST, and catalase, and lower levels of LPO in the cortex and hippocampus. Our present findings indicate that scopolamine causes oxidative stress in the cortex and hippocampus of rats, as shown by reduced anti-oxidant enzyme activity, while carveol has neuroprotective effects via decreasing oxidative stress. Our results were in line with the findings of the other studies (44, 45): memory impairments caused by scopolamine are linked to the development of oxidative stress. 

In addition, neuronal death was higher in the scopolamine group, while it was lower in the carveol group. Reduced neuronal loss in the cortex and the hippocampal area was observed in the carveol-treated group, which was mediated via anti-oxidant and anti-inflammatory action. By signaling via toll-like receptors and advanced glycation end products, Aβ, which is generated by the cleavage of APP, forms aggregates that activate microglia. These receptors activate the NF-κB transcription factors, which cause the generation of reactive oxygen species and the development of inflammatory mediators like cytokines (46, 47). TNF-α is a key pro-inflammatory cytokine in Alzheimer’s disease (48). Aβ activates the transcription factor NF-κB, which in turn increases TNF-α production in microglial cells (49). COX-2 is also strongly expressed in neurons, and its expression correlates with the presence of A deposits and tau tangles (50). Overexpression of COX-2 in neurons led to neuronal cell death in animal models of Alzheimer’s disease by forming A plaques and producing free radicals, resulting in worsened cognitive impairments (51). IHC and ELISA were used to access Aβ, NF-κB, TNF-α, COX-2, IL-18, and PGE2 levels in the rat brain and hippocampus in order to further verify the findings. All of these markers were increased in both areas in the scopolamine group, while carveol decreased the expression of these markers in the cortex and hippocampus, indicating its anti-inflammatory action. Carveol exhibited anti-amnesic potential by reducing Aβ synthesis and anti-inflammatory effects by substantially suppressing NF-κB and TNF-α, resulting in reduced expression of pro-inflammatory cytokines, according to previous studies.

DPPH: 1,1-diphenyl, 2-picrylhydrazyl

## Conclusion

Scopolamine induced memory impairment and inflammation by activating various inflammatory mediators such as Aβ/NF-κB/TNF-α/COX-2/IL-18, and PGE2 along with oxidative stress. Carveol exhibits an anti-amnesic effect, mediated through anti-oxidant, Aβ inhibition, and anti-inflammatory pathways, demonstrating its therapeutic potential in memory impairment. 

## Authors’ Contributions

SS Conceptualized and designed experimental work, and conducted the experiment. KL Compiled the data and contributed to writing the manuscript, conducted *in vivo *anti-oxidant, ELISA, and immunohistochemistry assays. AK Supervised the whole study. 

## Conflicts of Interest

The authors declare no conflicts of interest.
